# Healthcare Provider Perspectives on HIV Cure Research in Ghana

**DOI:** 10.1155/2023/8158439

**Published:** 2023-05-31

**Authors:** Helena Lamptey, Benjamin Newcomb, Evelyn Y. Bonney, James O. Aboagye, Peter Puplampu, Vincent J. Ganu, Gloria Ansa, Joseph Oliver-Commey, George B. Kyei

**Affiliations:** ^1^Department of Immunology, Noguchi Memorial Institute for Medical Research, College of Health Sciences, University of Ghana, Accra, Ghana; ^2^Departments of Medicine and Molecular Microbiology, Washington University School of Medicine in St. Louis, 660 S. Euclid Ave, St. Louis, MO, USA; ^3^Department of Virology, Noguchi Memorial Institute for Medical Research, College of Health Sciences, University of Ghana, Accra, Ghana; ^4^Department of Medicine, University of Ghana Medical School, College of Health Sciences, University of Ghana, Accra, Ghana; ^5^Department of Public Health, University of Ghana Hospital, Legon, Accra, Ghana; ^6^LEKMA Hospital, Accra, Ghana; ^7^Medical and Scientific Research Center, University of Ghana Medical Centre, Accra, Ghana

## Abstract

**Introduction:**

Antiretroviral therapy (ART) has reduced mortality and improved life expectancy among HIV patients but does not provide a cure. Patients must remain on lifelong medications and deal with drug resistance and side effects. This underscores the need for HIV cure research. However, participation in HIV cure research has risks without guaranteed benefits. We determined what HIV healthcare providers know about HIV cure research trials, the risks involved, and what kind of cure interventions they are likely to recommend for their patients.

**Methods:**

We conducted in-depth qualitative interviews with 39 HIV care providers consisting of 12 physicians, 8 counsellors, 14 nurses, 2 pharmacists, 2 laboratory scientists, and 1 community advocate from three hospitals. Interviews were transcribed verbatim and coded, and thematic analysis was performed independently by two investigators.

**Results:**

Participants were happy about the success of current treatments and hopeful that an HIV cure will be found in the near future, just as ART was discovered through research. They described cure as total eradication of the virus from the body and inability to test positive for HIV or transmit the virus. In terms of risk tolerance, respondents would recommend to their patients' studies with mild to moderate risks like what patients on antiretroviral therapy experience. Participants were reluctant to recommend treatment interruption to patients as part of a cure study and wished trials could be performed without stopping treatment. Healthcare providers categorically rejected death or permanent disability as an acceptable risk. The possibility of finding a cure that will benefit the individual or future generations was strong motivations for providers to recommend cure trials to their patients, as was transparency and adequate information on proposed trials. Overall, the participants were not actively seeking knowledge on cure research and lacked information on the various cure modalities under investigation.

**Conclusion:**

While hopeful for an HIV cure, healthcare providers in Ghana expect a cure to be definitive and pose minimal risk to their patients.

## 1. Introduction

The total number of people living with human immunodeficiency virus (HIV) continues to grow worldwide due to new infections and increased longevity of infected persons leading to an increased incidence prevalence ratio (IPR). In Ghana, HIV prevalence is estimated at 1.3–1.6% of the population, with significant regional variations in disease burden [1]. The IPR of HIV in Ghana is estimated at 6, well above the global target of less than 3, highlighting ongoing need for HIV treatment. HIV can be effectively suppressed with antiretroviral therapy (ART) in most patients and significantly extends their lifespan [2]. However, both HIV infection and ART have long-term health effects. Notably, HIV patients have higher rates of certain cancers, cardiovascular diseases, diabetes, chronic kidney diseases, and liver damage, as well as opportunistic infections [3, 4]. Long-term ART also has several risks, including decreased bone density, bone marrow suppression, cardiovascular disease, arrhythmias, liver and gallbladder disease, dyslipidemias, and insulin resistance [5, 6]. In addition, the cost of lifelong treatment is not sustainable in most low-income settings where ART availability is still dependent on donor funding. Taken together, there exists significant need for HIV cure and cure research.

HIV cure research faces significant ethical and practical hurdles. First, HIV cure trials carry the inherent danger of potentially requiring patients to discontinue or temporarily stop thoroughly validated ART medications (analytical treatment interruption) and expose participants to risks of viral rebound and transmission to sexual partners. Second, the remarkable success of ART may mean that any cure method introduced should have minimal risks to be accepted by patients. Third, the complexity of the cure approaches and methods currently under investigation presents another challenge.

Cure research takes four broad approaches as follows: (i) The shock and kill approach (latency reversal), which seeks to reactivate the latent virus under the cover of ART, leading to death of the T cells harboring the virus either by viral cytopathic effect or immune clearance; (ii) immunotherapy such as broadly neutralizing antibodies and chimeric antigen receptors; (iii) gene therapy such as CRISPR/Cas9 to excise or disable the virus in the host genome; or (iv) block and lock approach which seeks to prevent reactivation of the virus even when ART is discontinued [7–12]. These approaches may provide treatments that differ in the range and nature of side effects, duration, and intensity of treatment and whether they will eradicate the virus (eradication cure) or lead to a long-term remission (functional cure). Due to these uncertainties, inputs from both patients and their caregivers on the design of HIV cure are needed.

Experiences from prior clinical trials in Africa have demonstrated the vital role of stakeholder buy-in and institutional support [13–15]. Ghana started ART in 2003, and UNAIDS figures in 2020 estimate that 62% of HIV patients in Ghana are currently receiving ART, according to WHO guidelines. Great strides have been made to deploy widespread ART in Ghana, and the idea of interrupting ART may not be tenable for many providers [16]. Ghana represents a unique opportunity for studying stakeholder attitudes because many providers were practicing prior to the widespread use of ART and have participated in the ART revolution. Their experiences pre- and post-ART provide a unique perspective that will clarify the intricate social processes and provider behaviors that influence HIV cure research.

Few studies have specially evaluated the perspectives of patients or healthcare providers on HIV cure research, and these have been performed mostly in developed countries [17–21]. Since over 70% of people living with HIV are in Africa, it is critical to determine the attitudes of both patients and providers on the continent toward cure research, so that their inputs could be factored into cure designs and clinical trials. In this study, we performed in-depth interviews with 39 HIV healthcare providers to determine how they feel about the HIV cure, their outlook for a future cure, and what kinds of risks they are likely to recommend for their patients in future cure trials.

## 2. Methods

This stakeholder qualitative survey enrolled healthcare providers including doctors, nurses, counsellors, pharmacists, health educators, laboratory technicians, and community volunteers.. Thirty-nine providers were interviewed from the Korle-Bu Teaching Hospital, the largest HIV treatment center in Ghana; LEKMA hospital, a community hospital; and the University of Ghana Hospital which focuses on university students, staff, and their dependents. Given that participants were relatively homogenous in terms of interactions with HIV patients, we expected to attain saturation of the responses with 10–20 participants [22]. We interviewed 37 participants based on a convenience sampling at the three clinics. After written informed consent was obtained, interviews lasted for 20–30 minutes, and all recorded audios were de-identified and transcribed verbatim. The respondents were assured of the anonymity and confidentiality. Demographic information was obtained from each participant. The recorded interviews were transcribed word for word by the interviewers. The interviews were conducted in English, except for one interview that was conducted in the local dialect. The transcripts were read through by the authors, and deductive coding was used to identify the themes for data analysis. The deductive themes used include “whether there can ever be an HIV cure,” “ever searched for information on HIV cure,” “meaning or definition for HIV cure,” and “experience with patients on treatment.” Others were “sources of HIV cure and cures offered to the patients,” “perceived risks of participating in HIV cure trials” and “ the level of risks willing to accept for clinical trial,” and “motivation for encouraging patient participation in clinical trials related to HIV cure.” These themes were then analyzed using Excel software. After thematic codes were assigned to each interview question and the themes were categorized, it revealed common themes running through after about twenty interviews.

### 2.1. Ethical Statement

Ethical approval was obtained from the institutional review boards of Noguchi Memorial Institute for Medical Research (NMIMR-IRB-CPN 002/18-19), Ghana Health Service (GHS-ERC: 013/09/19), and the Korle Bu Teaching Hospital Ethical Review Board (KBTH-IRB/00075/2019).

## 3. Results

### 3.1. Demographic Characteristics of the Healthcare Providers

The median age of the providers interviewed was 34 years, while the median time of practice in their respective professions was 7 years (Table 1).

### 3.2. Whether There Can Ever Be an HIV Cure

Providers expressed the view that an HIV cure is imminent and was hopeful that this will happen in the near future or in their lifetime.

“*Yes, I am quite hopeful that we can have a cure. I don't expect a cure in the next five years, but I think within 10-15 years is a realistic possibility.” (Male, doctor)*.

*“Yes, we are hoping so. Because formally there was no medicine, but now we have medicines. So, I hope in the nearby future, we will have a cure.” (Female, community advocate)*.

Some of the respondents were of the view that just as there was no treatment for several illnesses in the past and research led to the development of potent drugs, they believe there will be a cure for HIV.

“*Yes, I think over the past years, there have been illnesses that had no cures, but then, with upcoming research and new discoveries, I think we have had cures to some of the illnesses. So, I think there could be a cure for HIV.” (Female, counsellor)*.

### 3.3. Knowledge on the Developments in HIV Research

Healthcare providers were asked if they have deliberately searched for any information on HIV cure. Some of the participants said they have performed that occasionally in the line of duty mainly to get information to answer patients' questions about HIV cure.

“*Yes. Once a while, I read on it online. Yes, I google and read on it”. “Not very often, but I have, because most times patients will keep asking you if there will ever be a cure or if there is a cure or they've heard about a supposed cure somewhere. So maybe you go and look it up on the net and then read about it, so I've done that a few times.” (Female, nurse)*.

The responses indicated that some of the HIV healthcare providers have never deliberately searched for information on HIV cure, though they have heard about cure research in the media, meetings, and conferences.

“*I've not really searched for a cure for HIV. I've not done that. Once a while, I read about the ARTs, but I've not really read about a cure.” (Female, nurse)*.

“*I've searched on some cures. Yeah. I did a little bit of read up on it. I think my most recent one was I think a few days or last week just brushing through, that the second person in the world has been cured.” (Male, doctor)*.

Healthcare providers were asked if they are aware of any approach researchers are using for HIV cure. Although some of the providers were aware that there is ongoing research towards HIV cure, they were not familiar with the specific studies. In general, respondents lacked knowledge on HIV cure approaches except for what is reported in popular media such as the few patients who have been cured through bone marrow transplant as indicated by the responses below:

“*The current plan I know was something about a stem cell that's being used, and the patients have been free for almost like a year.” (Female, doctor).*

*“And then I've also read about the second person who claimed to have been cured in the UK through stem cell transplantation for hematological malignancy. Well, I know that there's research into that, but not as a first-line treatment for HIV” (Female, doctor)*.

### 3.4. Healthcare Providers Definition of HIV Cure

Concerning their understanding of what a cure means, healthcare providers defined an HIV cure as eradication of virus from the blood of the patient such that the person does not have to take medications. Others said they expect a cured person to have undetectable virus and cannot transmit.

“*Cure will have to come with the virus not being detected, so the person wouldn't be on medication, and the person wouldn't be coming to see the doctor or having any form of symptoms even after the virus cannot be detected. That is what I will be looking out for.” (Female, nurse).*

***“ ****For me to be cured means that you do not have any count of the virus in you anymore and you cannot transmit and then you test negative to HIV tests.” (Male, doctor).*

*“I think if they don't have to take medications again and not transmitting HIV. No virus, even if they don't take drugs.” (Female, doctor)*

### 3.5. Experience with Patients on HIV Treatment

Healthcare providers shared their experiences about patients on ART and indicated that in general when patients are compliant, they get better and look healthy. The following quote summarizes this prevalent view:

“*It's been interesting, I've seen the progress. Some people come in here very weak, looking very sick, and actually very sick. And then later, they come in and they're strong, they've gained weight. It's encouraging to see patients getting better than they came in. So generally, it's good seeing the patients get better.” (Female, pharmacist)*.

On the other hand, some patients remain in denial, face psychological problems or feel stigmatized, and stop regular use of medication. This causes deterioration in health and sometimes loss of life.

“*So, I feel like most of them are not very compliant because they have medications that they have to take every day at specific times, which is quite difficult. You realize that most of them when they come to clinic, they are very down, they are unhappy. They find it difficult to disclose their status to people around them. So, I feel like the stigma is still there.” (Female, counsellor)*.

### 3.6. Claims of HIV Cure Remedies in Ghanaian Society

In Ghana, it is not uncommon to find people on radio or at the marketplace claiming to have cures for all kinds of diseases including HIV. The healthcare providers were asked if they have been approached and offered other forms of medications to try on their patients. Providers have heard of claims of HIV cure from traditional healers, herbalists, and spiritual healers. Although they have been approached with all sorts of cure remedies to offer to their patients, none of them had ever tried to do so.

“*Yes, we have a lot in the system. Mostly, they go on the herbs, and then they come back with either a kidney or liver failure, and then they just pass away.” (Female, nurse).*

*“We hear them on radios, from patients and the society. They talk about them a lot. And for the few times that I have experienced patients going to follow those cures, they come back worse. Some even come with conditions which may have been prevented. So they are in the system, but they don't help patients”. (Female, counsellor).*

*“We've had some people telling clients that they've prayed for them, and they're cured. So, we still have some people who will go to churches, come back, and say I want to retest. We test and they're still positive. Some will go and stay there and default and come back in a worse state, and then they'll have to restart ARV's.” (Female, pharmacist).*

*“A lot of them. We have a lot of our patients trying to seek for other remedies, but they always come back in an unfortunate situation. Most times, especially for the traditional healers, they will administer stuff because they are herbal, then the patients come back with organ failure and other things.” (Male, doctor).*

### 3.7. Level of Risk Providers Is Willing to Recommend to Their Patients during Cure Trials

When asked to mention what risks they perceive for HIV cure trials, participants talked about sociocultural risks, time commitment, side effects, organ damage, and death. Healthcare providers were willing to accept minimal risk such as mild headaches, diarrhea, skin rash, or vomiting for their clients to participate in clinical trials. One participant stated as follows:

“*But I don't think they will give it the go ahead if the risk is going to outweigh the benefit. If the trial is going ahead that means the benefit outweighs the risk. A very low risk. Zero to two level of risk out of 10.” (Female, nurse)*

Healthcare workers would not accept risk of any clinical trial that may lead to serious bodily harm or organ damage, serious sickness, or death. More importantly, they were concerned about trials that will lead to the interruption of ART for clients as exemplified in the quotes as follows:

“*I'm hoping that maybe in a way, there can be some sorts of trials that will not involve stopping their medications.” (Female, doctor).*

*“Yeah, stopping ARVs and the virus coming back is a huge risk to take, especially because we know that once they stop for some time, the viral load will go up again.” (Female, nurse).*

*“A few symptoms like fever, vomiting, but that's the most acceptable risk. But any organ damage wouldn't be acceptable.” (Male, doctor).*

*“I think you will be guided by the basic ethical principles. In terms of the risk, the risk will not be higher than the regular ARVs. If the risk is similar or less, I would be happy to take that risk” (Male, doctor)*.

### 3.8. Healthcare Providers' Willingness and Motivations to Recommend Trials to Patients

Most of the stakeholders are willing to encourage their patients to be involved in HIV cure clinical trials if they are sure there will be minimal risk, and the trial could result in finding a cure for their clients or the next generation of HIV patients.

“*I will encourage my patients to partake, because in the long run it will benefit them, yes, most importantly them. Then it also benefits the next generation.” (Female, counsellor.)*

Others were of the view that the health and management of HIV patients can be improved through research. For instance, safer medications that works more efficiently can be manufactured and used with minimal or no side effects. Therefore, they will still encourage their patients to participate in cure research.

“*I will still encourage them. Research is good, there are always new findings.” (Male, counsellor)*.

Concerning what will motivate stakeholders to encourage their patients to participate in a clinical trial relating to HIV cure, providers stated that the possibility of finding a cure that will benefit the individuals or future generations is paramount.

“*What will motivate me is the younger children I mentioned, their chances of having a cure and living medication-free for the rest of their lives.” (Male, counsellor).*

*“To help future generations. And just to relieve them of the daily medications that they are going through.” (Female, counsellor).*

*“The fact that it would lead to or contribute to that knowledge to help find a cure, they may not benefit themselves, because it may take a longer time. But, just that idea, that it could happen one day, that would make me encourage them to be a part of it.” (Female, doctor)*.

Healthcare providers also indicated that if the well-being of the patient is guaranteed in a clinical trial, then they will be motivated to encourage them to participate. They want a situation in which the benefits outweigh the risk involved in participation. They also want adequate information about the trials so that patients can make informed decisions.

“*So, if I think the risk is something that will be so much then I will be discouraged but will weigh the benefits against the disadvantages before I make a recommendation.” (Female, pharmacist).*

*“If I have additional information like preliminary studies done on product A showing a lot of promise, less side effects… oh yes of course.—because you see, we want cure, but we don't want to harm our patients.” (Male, doctor).*

*“Yes, and if enough information is given to us, then I will encourage them.” (Male, counsellor)*.

Generally, what will demotivate them from encouraging patients to participate in HIV cure related trails is when they perceive that their patient's lives may be at risk during a clinical trial.

“*I think the side effects. We know our patients are going through a lot, so if they are to be exposed to a clinical trial and go through more side effects, I think it will not help them.” (Female, nurse).*

*“You know, if the patients who volunteer to do the trial have adverse effects or no proper procedure is in place to take care of them. Or when the patients call for information and the PI or study coordinators are not providing the needed information, then I think that is a “no” for me.” (Male, doctor).*

*“I mean if there's a lot of harm involved, then that would be a demotivator.” (Female, doctor)*.

## 4. Discussion

In this study, we found that HIV care providers were optimistic that a cure will be found, considered a cure as total elimination of the virus from the body and were open to encouraging their patients to take part in future trials. However, given the success of ART, providers were not willing to encourage patients to take substantial risks such as analytical treatment interruption and would want investigators to be open and provide adequate information for all trials.

We observed that although healthcare providers were aware of ongoing research aimed at HIV prevention and ART drug trials, the level of awareness about HIV cure research was low. Those who were aware of ongoing research toward HIV cure did not have detailed knowledge of the various approaches being used. This low level of awareness of HIV cure research is similar to a study conducted on stakeholder perspective on HIV cure research in South Africa [23]. This underscores the need to educate all stakeholders on HIV cure research, since they will be instrumental in engaging patients who will be involved in such trials.

What constitutes an HIV cure is not clear, hence the emergence of the concepts of eradication or functional cure. For the participants in this study, they were unanimous that a cure means the patient has no virus in the body, tests negative for HIV, cannot transmit the virus, and does not have to see a doctor for HIV follow-up. This is a high bar for cure that researchers need to take note of as they design cure modalities. The expectation of complete viral elimination is not unique to Ghanaian healthcare providers as patients and providers in other settings find it difficult to grasp the concept of functional cure [24]. This means that more studies using robust methods such as choice experiments and human centered designs are needed to explore the concept of an HIV functional cure in both patients and providers.

Another issue that the respondents were unanimously concerned about is the likelihood of analytical treatment interruption during cure trials. Since there is no reliable biomarker for viral rebound or cure, ATI remains an integral part of cure trials. Since ATI carries the risk of viral rebound and transmission to sexual partners [25, 26], it is an important ethical issue that requires education and buy-in from stakeholders. Guidelines for ATI must be adhered to and explained well to both patients and their providers [27, 28]. Studies performed in the USA and France show that a majority of patients are willing to undergo ATI [18–20, 29, 30], which contrasts with studies performed in Africa [23, 31]. It will be interesting to study why these differences exist. We speculate that most patients and caregivers saw the devastation of the disease, have seen the impact ART, and have cognitive dissonance about the concept of ATI.

We found that the motivation for stakeholders to encourage their patients is based mainly on the possibility of finding a cure that will help future generations. Our results also suggest that healthcare provider buy-in is contingent on safely conducted trials that limit harm to patients. Respondents from our study cited safety concerns over side effects, fear of logistical challenges, possible social discrimination, general distrust of researchers, and requirements for information sharing with providers. These fears probably stem from general distrust of researchers and call for more education of healthcare providers in research ethics and methods. More importantly, the providers themselves should be involved in the design of the trial, so they can be confident to recommend it to their patients. These concerns were in line with previously published literature [32–34]. Risk tolerance among Ghanaian healthcare providers was found to be relatively low because their comparator for a cure trial is current ART which has minimal side effects. Therefore, the success of ART sets a high bar for future agents that will be tested for HIV cure.

There were some limitations to our study. We selected participants based on their availability in the three HIV clinics which may introduce self-selection bias. Our study had about twice the number of female providers compared to male which mirror what is seen in the clinics. In our analysis, we did not notice different trends between females and males. We observed that similar responses emerged halfway after thematic codes were assigned; thus, thematic saturation might have been achieved as mentioned in previous studies [22, 35]. Other limitations were interviewer bias and respondent reluctance to speak freely. Interviewer bias was minimized by using structured analysis tools that have been previously validated in the social sciences literature. Specifically, the coding of themes and the use of Structured Analytics software helped to reduce analytic bias. Overcoming respondent reluctance to speak freely was alleviated by de-identification of all responses and using techniques such as probing and the use of hypothetical examples to assess attitudes.

In summary, our results suggest that healthcare providers are hopeful for an HIV cure but obtaining provider buy-in will require transparency on the part of investigators, as well as cure modalities that are likely to have benign side effects.

## Figures and Tables

**Table 1:** Demographic characteristics of the healthcare providers in the survey.

Healthcare provider	Total number (39)
Age median (range)	34 years (26–59)

Gender (%)	*n* (%)
Male	12 (30.8)
Female	27 (69.2)

*Specialty (%)*	
Doctors	12 (30.8)
Counselors	8 (20.5)
Nurses	14 (35.9)
Pharmacists	2 (5.1)
Lab scientists	2 (5.1)
Community advocate	1 (2.6)

Years in practice median (range)	7 (1–21 years)

## Data Availability

The data that support the findings of this study are available on request from the corresponding author. The data are not publicly available due to privacy or ethical restrictions.
